# Effect of Nephropathy Prescription I on the Expression of Angptl3 and Podocyte-Associated Protein in Mice with Adriamycin-Induced Nephropathy

**DOI:** 10.1155/2022/9921679

**Published:** 2022-01-31

**Authors:** Feifei Zhang, Junchao Liu, Jian Yu, Wen Sun, Yonghong Wang, Teng Fan, Yanyan Sun, Xinghui Han

**Affiliations:** Children's Hosptial of Fudan University Department of Traditional Chinese Medicine, Shanghai 201102, China

## Abstract

**Objective:**

This study aimed to investigate the effects of Nephropathy Prescription I on the expression of angptl3, nephrin, and podocin, in addition to its protective effects on podocytes in mice with adriamycin-induced nephropathy.

**Methods:**

BALB/c mice were randomly divided into the control (C), adriamycin (Model or M), adriamycin + Nephropathy Prescription I (*M* + *Z*), adriamycin + prednisone acetate (*M* + *S*), and adriamycin + Nephropathy Prescription I + prednisone acetate groups (*M* + *Z* + *S*). All mice except those in the C group in the experimental groups were treated with a single tail vein injection of adriamycin. The urine albumin-creatinine ratio was measured before model establishment and on the 7th day, 14th day, 21st day, and 28th day of doxorubicin injection. All the mice were sacrificed on the 29th day. Blood samples were collected to observe biochemical indicators in the serum. The morphological structure and podocyte ultrastructure in the kidney were observed using light and electron microscopy, respectively. The expression of angptl3, nephrin, and podocin at the mRNA and protein levels was detected by real-time PCR and western blotting, respectively.

**Results:**

Following modeling with adriamycin, albuminuria was observed in urine samples in the first week, and the urinary protein/creatinine ratio increased maximally in the fourth week in the *M* group (*P* < 0.05). In contrast, the urinary protein/creatinine ratio significantly decreased (*P* < 0.05) in the third week in the (*M* + *Z*) group compared to that in the *M* group. Similarly, this ratio decreased in the (*M* + *S*) and (*M* + *Z* + *S*) groups compared to that in the *M* group throughout the experiment. Compared with the C group, serum albumin content and the expression of nephrin and podocin decreased (*P* < 0.05), whereas blood lipid level and the expression of angptl3 increased (*P* < 0.05) in the *M* group. Glomerular foot process fusion was observed in this group using electron microscopy. In all the intervention groups, serum albumin content and the expression of nephrin and podocin increased (*P* < 0.05), whereas blood lipid level and the expression of angptl3 decreased (*P* < 0.05), with alleviated glomerular foot process injury observed particularly in the (*M* + *Z* + *S*) group.

**Conclusion:**

The Nephropathy Prescription I can alleviate albuminuria, increase serum albumin levels, lower blood lipid levels, and reduce the fusion of foot processes of podocytes in mice with adriamycin-induced nephropathy. The protective effects of the Nephropathy Prescription I may function by reducing Angptl3 expression and increasing nephrin and podocin expression.

## 1. Introduction

Primary nephrotic syndrome (PNS) is a common kidney disease in children. The main clinical manifestations are proteinuria, hypoproteinemia, hyperlipidemia, and edema. It has a prevalence of 12–16 per 100,000 children, with an annual incidence of 2–7 per 100,000 children. A large amount of proteinuria is the most characteristic clinical manifestation and an independent risk factor directly affecting the progression of renal disease [1, 2]. Nephrotic syndrome during childhood mainly manifests with minimal pathological changes, accounting for approximately 80% of primary nephrotic syndrome [3]. Kidney diseases in children have their own characteristics as in adults, and most of the patients with kidney diseases respond well to hormone therapy. Therefore, hormone therapy is the first choice for the treatment of kidney diseases in clinical practice. However, a considerable number of children frequently relapse and even display resistance to hormone therapy. In addition, long-term hormone therapy may have a series of side effects such as cataracts, hypertension, osteoporosis, and adverse effects on the growth and development of children and adolescents. Treatment of childhood nephrotic syndrome with hormones and/or immunosuppressants and biologics also has certain toxic and side effects.

Nephrotic syndrome belongs to the category of “edema” in Chinese medicine. Chinese medicine has a history of thousands of years in the diagnosis and treatment of kidney diseases. Traditional Chinese medicine (TCM) has a long history of being used for treating kidney diseases. Modern Chinese medicine especially emphasizes the combination of disease differentiation and syndrome differentiation. It follows the law of treatment based on syndrome differentiation and integrates Western medicine to achieve the best therapeutic efficacy. Nephropathy Prescription I is an in-hospital TCM formula in the Department of TCM of the Children's Hospital of Fudan University, based on classic ancient prescriptions such as “Poria Five Powder” and “Shenqi pill.” This prescription is usually used in childhood nephrotic syndrome with spleen and kidney yang deficiency or lung and spleen qi deficiency, before hormone initiation, after withdrawal, or for dose reduction followed by proteinuria. Nephropathy Prescription I alone or combined with hormone and immunosuppressive agents can reduce the resolution of edema, increase serum albumin, reduce blood lipids, and reduce hormonal side effects. Although Nephropathy Prescription I has a certain effect, the underlying mechanisms of this formula are unclear.

In a mouse model with adriamycin-induced nephrotic syndrome, we investigated the effects of Nephropathy Prescription I on the biochemistry, pathology, and podocyte-related protein levels, providing evidence for its mechanism of action in treating children with nephrotic syndrome.

## 2. Materials and methods

### 2.1. Materials

#### 2.1.1. Animals

Fifty 6–8 weeks old male BALB/c mice weighing 20–25 g were purchased from Slack Laboratory Animal Co., Ltd. (SCXK (Shanghai) 2017–0005) and housed in the Experimental Animal Center of Shanghai University of Traditional Chinese Medicine (SCXK (Shanghai) 2014–0008). Animals had free access to food and water and were kept in an environment at 20 ± 2°C for one week prior to the experiment. Mice maintained normal activity, with shiny fur, no hair loss, normal feeding, and negative urine protein.

### 2.2. Agents

Adriamycin (D1515, 10 mg) was purchased from Sigma; prednisone (5 mg) was obtained from Sinepharm (H31020675, Shanghai, China); antibodies against nephrin (Novus, NBP1-30130), podocin (Novus, NBP2-26057), and angptl3 (RnD, AF136) were used.

### 2.3. TCM Preparation

Nephropathy Prescription I was composed of 15 g Radix Astragali, 3 g Cassia Twig, 9 g *Atractylodes macrocephala*, 9 g *Alisma*, 9 g *Curculigo*, 9 g *Epimedium brevicornum*, 9 g Poria, 9 g *Glycyrrhiza*, and 15 g *Euryale ferox* (Shanghai Hongqiao TCM).

Nephropathy Prescription I was prepared using the classic water extraction and ethanol sedimentation method [4, 5].

## 3. Methods

### 3.1. Animal Studies

Animals were randomly divided into five groups (*n* = 10): control group (C), adriamycin group (M), adriamycin + Nephropathy Prescription I group (*M* + *Z*), adriamycin + prednisone acetate group (*M* + *S*), and doxorubicin + Nephropathy Prescription I + prednisone acetate group (*M* + *Z* + S). Nephrotic mice were established by tail vein injection of adriamycin (10 mg/kg) [6–11], while animals in the control group were injected with an equal amount of normal saline.

### 3.2. Drug Intervention

The Nephropathy Prescription I (26.1 g/(kg.d)) [12, 13] and prednisone (12 mg/(kg.d)) [14] were intragastrically administrated once a day for 4 weeks consecutively. On the 29th day, all the mice were anesthetized, then fixed and perfused and their kidneys were collected and fixed with 4% neutral formalin. Some samples were fixed with 3% glutaraldehyde for electron microscopy. Renal cortex samples were used for quantitative real-time polymerase chain reaction (PCR) and western blotting.

### 3.3. Detection of Indices

#### 3.3.1. Urine Protein/Creatinine

Urine samples were centrifuged, and the supernatants were collected. An enzyme-linked immunosorbent assay (ELISA) was used to detect the urine protein concentration using a Mouse Urinary Albumin Detection Kit (Chondrex). Creatinine was detected by the sarcosine oxidase method according to the manufacturer's instructions (Nanjing Jiancheng Institute of Bioengineering).

### 3.4. Serum Biochemistry (Table 1)

#### 3.4.1. Microscopy

Kidney samples were embedded in paraffin, sectioned into 4 *µ*m slices, and subjected to hematoxylin and eosin (HE) staining. Kidney sections were also stained for periodic acid-Schiff stain followed by detection using an optical microscope.

### 3.5. Electron Microscopy

Tissue samples were fixed in 3% glutaraldehyde in 0.1 M phosphate buffer (PB, pH 7.4), 1% osmium acid in 0.1 MPB (pH 7.4), dehydrated in graded ethanol, and embedded in epoxy resin. Ultrastructural changes were observed using a transmission electron microscope (HITACHI, HT7700).

### 3.6. Quantitative Real-Time PCR

Total RNA from the renal cortex samples was extracted using TRIzol, reverse transcribed into cDNA, and amplified using SybrGreen reagent. Primers were designed using Primer Premier 5.0 and synthesized by Shanghai Qinxi Biotech (Table 2). PCR reactions consisted of an initial denaturing step (95°C, 20 min), followed by 40 cycles of denaturing (95°C, 30 s), annealing (30 s), extension (72°C, 40 s), in 2 × qPCR Mix (12.5 *µ*L), 7.5 *µ*M primers (2.0 *µ*L), cDNA (2.5 *µ*L), and ddH_2_O (8.0 *µ*L). All PCR reactions were conducted on a 7300 real-time PCR system (ABI, USA). The relative mRNA levels were normalized to that of the internal glyceraldehyde 3-phosphate dehydrogenase (Table 2).

### 3.7. Western Blot Analysis

Kidney tissues were cut into small pieces and ground in lysis buffer and a protease inhibitor. The supernatant concentration was determined, and 30 *µ*g of samples were loaded and separated using 10% sodium dodecyl sulfate-polyacrylamide gel electrophoresis. Proteins were subsequently transferred onto polyvinylidene difluoride membranes activated with methanol for 15 s. Thereafter, the membrane was blocked in 5% slimmed milk and incubated with the primary antibodies at 4°C overnight. Following washing with Tris-buffered saline with Tween, the membrane was incubated with the secondary antibody at room temperature for 2 h. Protein signals were detected using enhanced chemiluminescence kits.

## 4. Statistical Analysis

Quantitative data with normal distribution were expressed as $\bar{x}\pm s$ and analyzed with univariate analysis of variance, followed by the least significant difference. Else, the Kruskal–Wallis rank sum test was used followed by the Dunn–Bonferroni method. Repeated measures analysis of variance was used for repeated measures data. The SPSS 24.0 software was used for statistical analysis, and the difference was statistically significant at *P* < 0.05.

## 5. Results

### 5.1. Body Weight Changes

**Figure 1:**
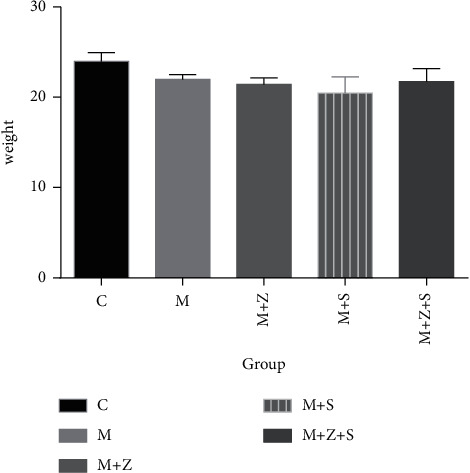
Changes in body weight (g) in different groups: control group (C), adriamycin group (M), adriamycin + Nephropathy Prescription I group (*M* + *Z*), adriamycin + prednisone acetate group (*M* + *S*), and adriamycin + Nephropathy Prescription I + prednisone acetate group (*M* + *Z* + *S*).

Changes in body weight in different groups over 4 weeks are shown in Table 3 and Figure 1, and the general condition of animals at day 28 is shown in Table 4.

### 5.2. Urine Albumin/Creatinine Ratio

**Figure 2:**
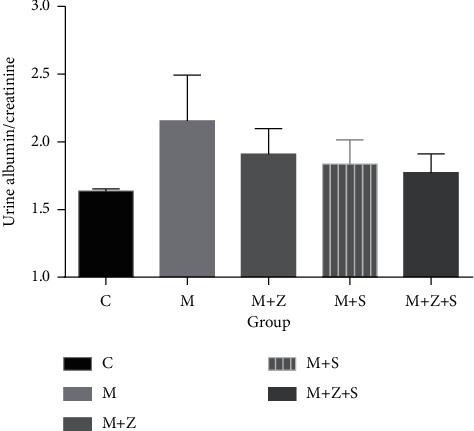
Changes of urine albumin/creatine ratio in different groups (mg/mg): control group (C), adriamycin group (M), adriamycin + Nephropathy Prescription I group (*M* + *Z*), adriamycin + prednisone acetate group (*M* + *S*), and adriamycin + Nephropathy Prescription I + prednisone acetate group (*M* + *Z* + S).

We observed urine albumin in the model group after treatment with adriamycin for weeks 1 and 2, and an elevated urine albumin/creatine ratio was observed compared to that of the control group (*P* < 0.05). The urine albumin/creatine ratio decreased in the (*M* + *S*) and (*M* + *Z* + *S*) groups compared to that of the model group (*P* < 0.05). At weeks 3 and 4, the urine albumin/creatine ratio in the *M* group was higher than that in the control group (*P* < 0.05). Compared to the *M* group, a decreased urine albumin/creatine ratio was observed in (*M* + *Z*) and (*M* + *S*) groups (*P* < 0.05) and especially in the (*M* + *Z* + *S*) group (*P* < 0.01) (Table 5 and Figure 2).

## 6. Serum Biochemical Indices

### 6.1. Serum Albumin

**Figure 3:**
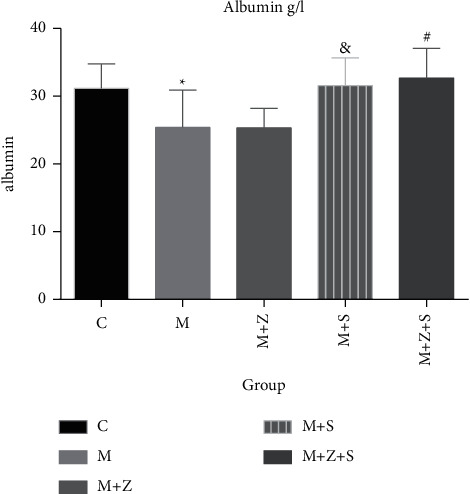
Changes in serum albumin in different groups (g/L): control group (C), adriamycin group (M), adriamycin + Nephropathy Prescription I group (*M* + *Z*), adriamycin + prednisone acetate group (*M* + *S*), and adriamycin + Nephropathy Prescription I + prednisone acetate group (*M* + *Z* + S).

Four weeks after adriamycin treatment, the serum albumin level in the model group decreased compared to that in the control group (*P* < 0.05). Compared to the model group, elevated serum albumin was observed in the (*M* + *Z*) and (*M* + *S*) groups (*P* < 0.05) and especially in the (*M* + *Z* + *S*) group (*P* < 0.01) (Table 6 and Figure 3).

### 6.2. Total Cholesterol (TC) and Triglyceride (TG)

**Figure 4:**
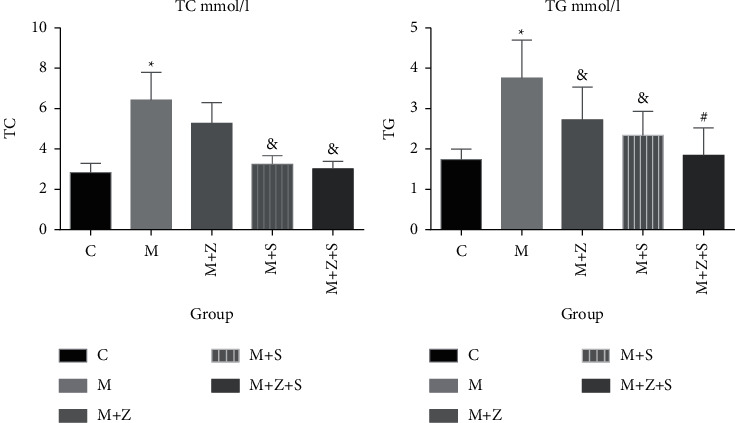
Changes in total cholesterol and triglyceride levels in different groups (mmol/L): control group (C), adriamycin group (M), adriamycin + Nephropathy Prescription I group (*M* + *Z*), adriamycin + prednisone acetate group (*M* + *S*), and adriamycin + Nephropathy Prescription I + prednisone acetate group (*M* + *Z* + S).

Four weeks later, the levels of TC and TG were elevated compared to the control group (*P* < 0.05), whereas the TC and TG content decreased in the (*M* + *Z*), (*M* + *S*) (*P* < 0.05), and (*M* + *Z* + *S*) (*P* < 0.05) groups compared to that in the model group (Table 7 and Figure 4).

## 7. Optical Microscopy and Electron Microscopy

### 7.1. Pathological Changes of Glomerulus under Optical Microscopy

**Figure 5:**
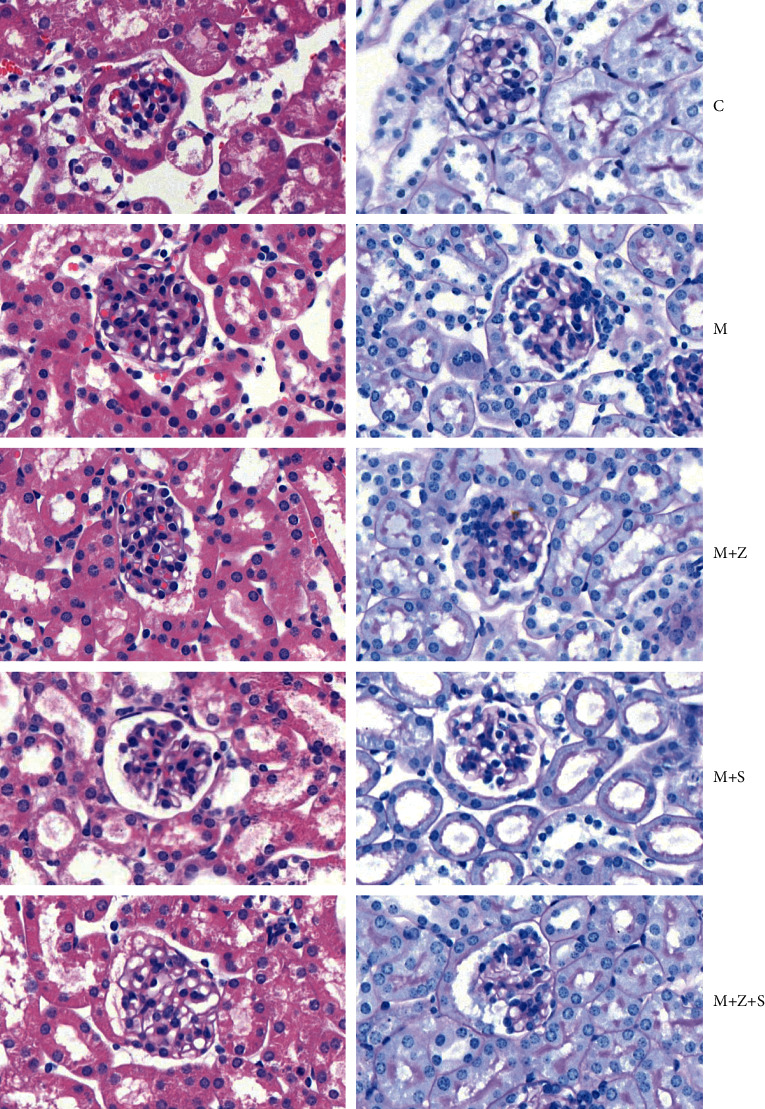
Pathological changes of glomerulus under optical microscopy (HE × 400, PAS × 400) in different groups: control group (C), adriamycin group (M), adriamycin + Nephropathy Prescription I group (*M* + *Z*), adriamycin + prednisone acetate group (*M* + *S*), and adriamycin + Nephropathy Prescription I + prednisone acetate group (*M* + *Z* + S).

There were no apparent pathological changes in the glomerulus under optical microscopy in different groups (Figure 5).

### 7.2. Electron Microscopy

**Figure 6:**
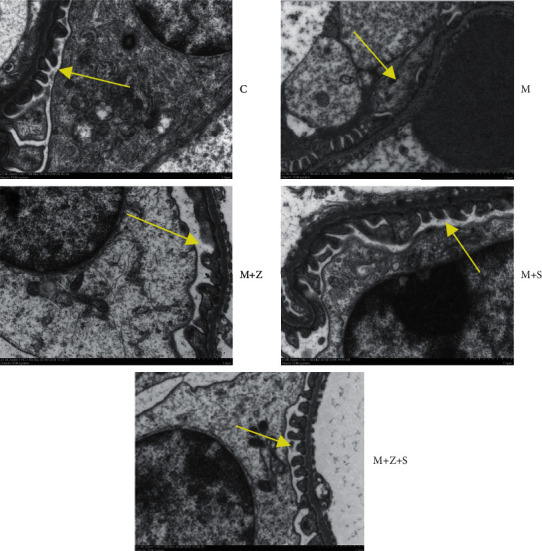
Electron microscopy images of morphological changes in foot processes in mice with nephropathy (× 7000) of different groups: control group (C), adriamycin group (M), adriamycin + Nephropathy Prescription I group (*M* + *Z*), adriamycin + prednisone acetate group (*M* + *S*), and adriamycin + Nephropathy Prescription I + prednisone acetate group (*M* + *Z* + *S*). There was no significant change in the structure of glomerular podocytes in the C group; the glomerular basement membrane in the M group was thickened, and foot processes were diffusely fused or disappeared; foot processes in the (*M* + *Z*) group were partially fused; foot processes were clearer in the (*M* + *Z* + S) group.

There was no significant change in the structure of glomerular podocytes in the C group; the glomerular basement membrane in the *M* group was thickened, and foot processes were diffusely fused or disappeared; foot processes in the (*M* + *Z*) group were partially fused; foot processes were clearer in the (*M* + *Z* + *S*) group (Figure 6).

### 7.3. mRNA Levels of Nephrin, Podocin, and Angptl3 in Different Groups

**Figure 7:**
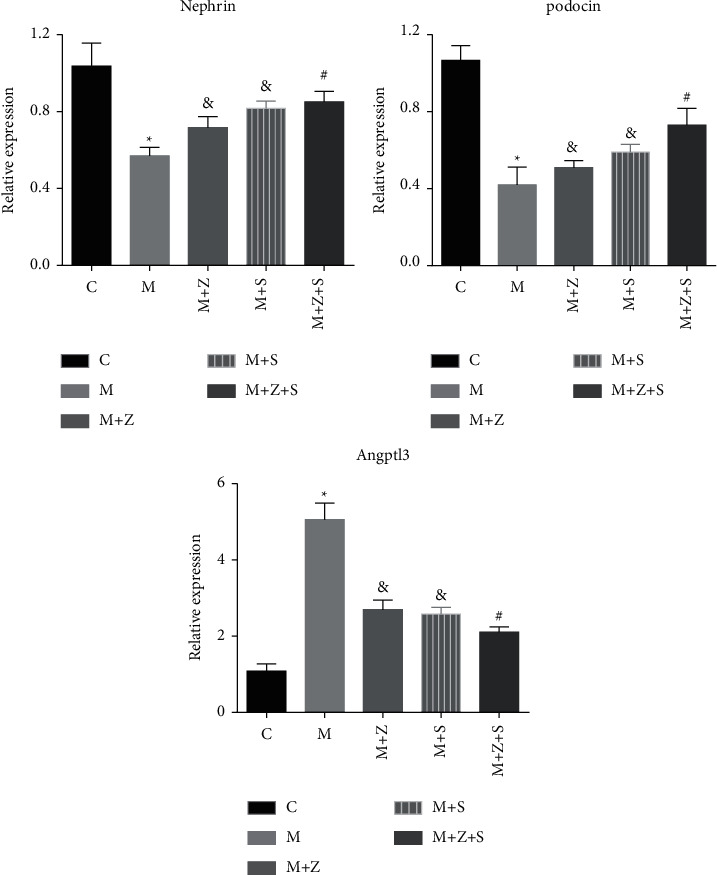
mRNA levels of nephrin, podocin, and Angptl3 in different groups. ^*∗*^M vs. C group (*P* < 0.05); ^&^*M* + *Z* or *M* + *S* vs. M group (*P* < 0.05); ^#^*M* + *Z* + S vs. M group (*P* < 0.01).

Four weeks after adriamycin treatment, mRNA levels of nephrin and podocin decreased, while Angptl3 mRNA levels were higher in the model group than in the control group (*P* < 0.05). Compared to the model group, mice in the (*M* + *Z*) (*P* < 0.05) and (*M* + *S*) (*P* < 0.05) groups, and especially the (*M* + *Z* + S) group (*P* < 0.01), exhibited elevated mRNA levels of nephrin and podocin and decreased Angptl3 levels (*P* < 0.05) (Figure 7).

### 7.4. Protein Levels of Nephrin, Podocin, and Angptl3 in Different Groups

**Figure 8:**
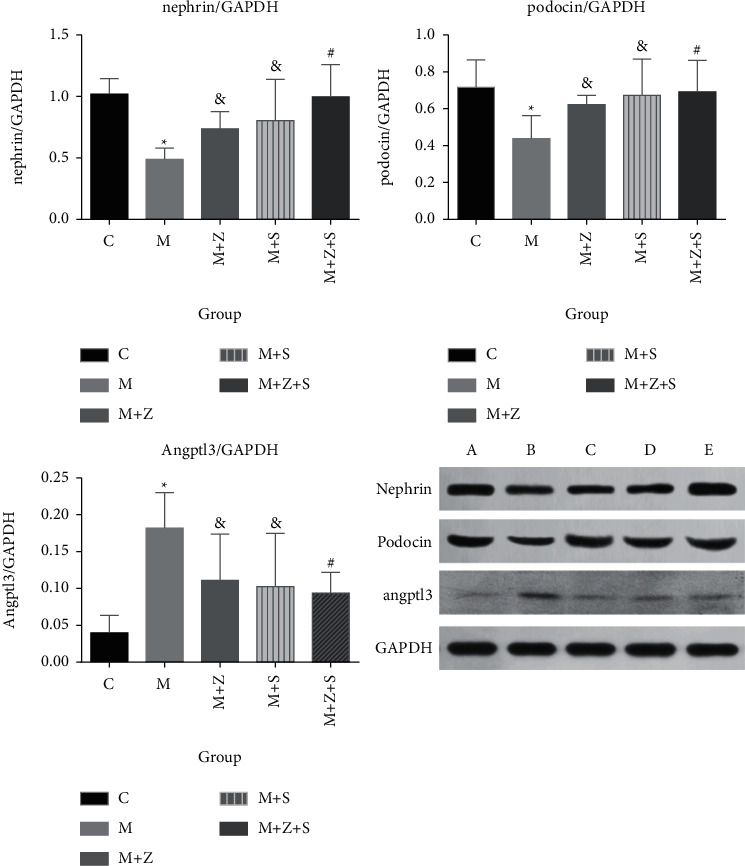
Protein levels of nephrin, podocin, and Angptl3 in different groups: (a) control (C); (b) model group (M); (c) traditional Chinese medicine (TCM) group (*M* + *Z*); (d) (*M* + *S*); and (e) (*M* + *Z* + S). ^*∗*^M vs. C group (*P* < 0.05); ^&^*M* + *Z* or *M* + *S* vs. M group (*P* < 0.05); ^#^*M* + *Z* + S vs. M group (*P* < 0.01).

Four weeks after adriamycin treatment, protein levels of nephrin and podocin decreased, while Angptl3 levels increased in the model group compared to those in the control group (*P* < 0.05). Compared to the model group, mice in the (*M* + *Z*) (*P* < 0.05) and (*M* + *S*) (*P* < 0.05) groups, and especially in the (*M* *+* *Z* *+* *S) (P* < 0.01) group, exhibited elevated protein levels of nephrin and podocin and decreased Angptl3 levels (*P* < 0.05) (Figure 8).

## 8. Discussion

Nephrotic syndrome belongs to the category of “edema” in Chinese medicine. Deficiency is an important pathogenesis of proteinuria, which may cause damage to the spleen, lungs, and kidneys. Moreover, deficiency could lead to loss of essence, energy, blood gas, and yin and yang. Loss of innate and acquired kidney essence may cause proteinuria. Damp heat and blood stasis may also aggravate proteinuria.

Insufficient natural endowment, chronic illness, physical weakness, and external pathogenic factors can cause dysfunction of the lung, spleen, and kidney, dysfunction of qi, storage imperfection, essence leakage, and liquid stagnation, resulting in proteinuria.

Nephropathy Prescription I is composed of Radix Astragali, Cassia Twig, *Atractylodes macrocephala*, *Alisma*, *Curculigo*, *Epimedium brevicornum*, Poria, *glycyrrhiza*, and *Euryale ferox*, and based on Poria Five Powder and Shenqi pill. This concoction is frequently used in the Department of Traditional Chinese Medicine Nephropathy in Children's Hosptial of Fudan University Department, with good efficacy for childhood nephrotic syndrome with spleen and kidney deficiency, especially in children with low-dose hormone-dependent and frequent proteinuria. One of the primary components of this prescription, the Huangqi Poria Five Powder, effectively reduced proteinuria, increased serum albumin, and decreased blood lipids in a clinical study [15]. Moreover, it could alleviate adriamycin-induced proteinuria, edema, and hyperlipemia in animal models, with an enhanced effect when combined with hormones rather than hormone therapy alone [16]. In 2012, Lei et al. found that Wulingsan combined with Western medicine effectively reduced the resolution of edema and recovered serum and urine proteins, and also reduced hormone-induced side effects in primary nephrotic syndrome [17].

Nephrotic syndrome is a common disease of the urinary system in children, and the course of the disease in some children is prolonged and easy to repeat, which seriously affects children's health [18]. According to domestic statistics, this disease is second only to acute nephritis among hospitalized children due to urinary system diseases [19]. Nephrotic syndrome is characterized by a large amount of proteinuria and minimal pathological changes. Electron microscopy revealed the disappearance of glomerular podocyte fusion. Podocyte injury plays an important role in nephrotic syndrome. Podocytes are located in the outermost layer of the glomerular filtration barrier. The dynamic hiatus diaphragm formed by interlacing podocytes' foot processes ensures that podocytes can feel and adapt to the changing environment and maintain the selective filtration function of the glomerular [20].

The occurrence of proteinuria is closely associated with podocytes. Damaged split podocyte membranes can cause protein leakage from the kidney, leading to proteinuria. The slit membrane is composed of a variety of proteins, including podocin and nephrin [21].

Nephrin is a transmembrane protein expressed in glomerular podocytes. Early podocyte structural changes are characterized by separation from the glomerular basement membrane. Persistent pathology leads to severe and progressive glomerular damage [22].Podocin is a transmembrane protein of glomerular podocytes and is abundantly expressed in the kidney, which is structurally associated with nephrin [23]. Podocin deregulation affects podocyte foot processes, the integrity of the slit membrane, and the filtration function of the glomerulus.

Studies have shown that Huaiqihuang granules can upregulate the expression of nephrin and podocin mRNA in the podocytes of adriamycin nephropathy rats and maintain the integrity of the podocyte hiatus diaphragm [24, 25]. Qufeng Tongluo prescription can improve proteinuria in adriamycin nephropathy rats, upregulate the expressions of nephrin and podocin in podocytes, alleviate fusion of foot processes, and alleviate damage of podocytes [26–28]. Shenqi Dihuang decoction can reduce proteinuria, alleviate podocyte damage, and increase nephrin expression in adriamycin nephropathy mice [29]. After astragaloside IV intervention in rats with adriamycin nephropathy for 12 weeks, the expression of nephrin and podocin proteins related to podocytes in the intervention group increased. Astragaloside IV maintained diaphragm integrity and reduced proteinuria levels in rats with adriamycin-induced nephropathy [30].

With the deepening of research, more newly discovered podocyte proteins are involved in podocyte damage. Persistent expression of Angptl3 in the kidney was first reported in 1999 [31]. Angptl3 regulates lipid metabolism by inhibiting LPL (lipoprotein lipase) activity. There was no disulfide bond between Angptl3 molecules, but there were still large Angptl3 molecules. Angptl3 was mainly distributed in the liver and regulated by LXR (liver *X* receptor), and the expression of Angptl3 was not affected by fasting [32]. Angptl3's C-terminal FNB domain binds to integrin alpha (V) beta(3) and activates integrin alpha (V) beta(3) and Akt phosphorylation-mapk-FAK signaling pathway, leading to adhesion and migration of liver endothelial cells. At the same time, studies in vivo found that the C-terminal FNB domain of Angptl3 had the role of angiogenesis [33]. In 2013, Vickers et al. screened microRNA (miRNA)-27b and found that it can specifically regulate the expression of Angptl3 [34], suggesting that exogenous administration of mirNA-27b may be a research method to reduce the expression of Angptl3. Currently, Angptl3 is considered as a new potential target for the treatment of metabolic syndrome [35]. It has the function of regulating lipid metabolism [36, 37], which is related to the secretion and function of insulin [38, 39] and can also affect stem cell proliferation [40].

High-throughput sequencing data revealed elevated expression of Angptl3 in kidney tissues of patients with nephrotic syndrome from more than 18,000 genes. Further studies found elevated mRNA levels of Angptl3 in the glomeruli of children with kidney disease compared to normal controls, and higher expression of Angptl3 in minimal change disease [41]. Further investigation suggested that Angptl3 in the glomeruli was positively correlated with the degree of foot process fusion of podocytes and the ratio of urine protein/creatinine [42]. Later, a research study found that the expression of Angptl3 in kidney tissue and serum of an adriamycin rat model was increased, and the expression of Angptl3 in kidney tissue was increased with the increase of urinary protein [43, 44]. Podocytes cultured with purinomycin in vitro can secrete Angptl3 in large quantities and rearrange cytoskeleton. The expression of Angptl3 increased in a time-dependent manner and increased with the concentration of purinomycin [45, 46]. The results of the study showed that the high expression of Angptl3 in podocytes is involved in podocyte damage. Therefore, actively looking for drugs that inhibit the expression of Angptl3 is a key step in the implementation of the strategy of protecting podocytes.

In this study, minimal change nephropathy in mice was established through tail vein administration of adriamycin (10 mg/kg), and urine samples were collected every week. The data showed that the urine albumin/creatinine ratio gradually increased and peaked at week 4. Meanwhile, mice exhibited decreased serum albumin and increased serum lipids, and foot process fusion was detected using electron microscopy, suggesting the successful establishment of a nephrotic animal model. Intervention with TCM or prednisone could alleviate the degree of proteinuria, increase serum albumin levels, and decrease blood lipids. Such effects were much more evident in the TCM + prednisone group. The efficacy of TCM treatment alone was not as good as that of the combined treatment. Electron microscopy indicated that intervention with TCM or prednisone could alleviate the degree of podocyte foot process fusion, with the highest effect observed in the TCM plus Western medicine group. These results suggest that Nephropathy Prescription I can reduce urine protein, increase serum albumin, decrease blood lipids, and protect podocytes in nephrotic mice.

For the first time, we evaluated the effects of Nephropathy Prescription I on the expression of Angptl3 in nephrotic animals. Compared to the control group, mRNA and protein levels of Angptl3 decreased in the model group and were elevated in the TCM intervention group. Moreover, the levels of nephrin and podocin were elevated in the model group and decreased in the TCM group. Collectively, these data suggest that Nephropathy Prescription I exhibited protective effects against adriamycin-induced nephropathy by regulating Angptl3, nephrin, and podocin.

This study proved that the therapeutic effect of TCM combined with Western medicine is better than that of treatment with TCM or Western medicine alone, consistent with clinical practice. The following questions require in-depth research in the future: Why is combined treatment with TCM and Western medicine better than TCM or Western medicine alone? What is the optimal regime of Nephropathy Prescription I and prednisone to improve efficacy with fewer side effects? What are the signaling pathways and components associated with its effect?

## Figures and Tables

**Table 1:** Serum biochemical indices.

Serum biochemical indices	Method	Kit
Serum albumin	ELISA	Mouse Albumin ELISA Kit (Bethyl Laboratories)
Total cholesterol and triglyceride	GPO-PAP	Nanjing Jiancheng Institute of Bioengineering

**Table 2:** Primers used in this study.

Gene	Primers (5′-3′)	Length (bp)	Annealing temperature (°C)
M-GAPDH-S	CCTCGTCCCGTAGACAAAATG	133	60
M-GAPDH-A	TGAGGTCAATGAAGGGGTCGT		60
M-podocin -S	AGAGCGAGCGACCAGAGGAA	115	60
M-podocin -A	AACCAGATGGAAAAAGGGAACG		60
M-nephrin-S	GCGGTTGGTGGTCTTCTGTTG	194	60
M-nephrin-A	GTGCTAACCGTGGAGCTTCTTG		60
M-Angptl3-S	ACTCCCCCTCTTCAACTGAAC	200	60
M-Angptl3-A	GAGCCATCTTTCCGGTGTTGA		60

**Table 3:** Changes in body weight (g) in different groups over 4 weeks.

Group	Time				
	Week 0	Week 1	Week 2	Week 3	Week 4
C group	22.96 ± 1.02	23.02 ± 1.13	24.23 ± 1.04	24.55 ± 0.95	25.15 ± 1.09
M group	22.61 ± 1.32	22.38 ± 1.15^▲^	21.87 ± 1.27^▲^	21.26 ± 1.29^▲^	21.79 ± 1.16^▲^
*M* + *Z* group	22.23 ± 0.81	22.13 ± 1.13^★^	21.19 ± 1.17	20.61 ± 1.20^★^	20.99 ± 1.25^★^
*M* + *S* group	23.10 ± 1.49	21.00 ± 1.27	20.64 ± 1.55	18.95 ± 1.18	18.57 ± 2.79
*M* + *Z* + *S* group	23.79 ± 0.98	22.35 ± 1.15^#^	21.80 ± 0.84^#^	20.29 ± 0.45^#^	20.44 ± 0.88^#^

Control group (C), adriamycin group (M), adriamycin + Nephropathy Prescription I group (*M* + *Z*), adriamycin + prednisone acetate group (*M* + *S*), adriamycin+ Nephropathy Prescription I + prednisone acetate group (*M* + *Z* + *S*). ^▲^*M* + *S* vs. M group at the same time point (*P* < 0.05); ^★^*M* + *S* vs. *M* + *Z* at the same time point (*P* < 0.05); ^#^*M* + *S* vs. *M* + *Z* + *S* at the same time point (*P* < 0.05).

**Table 4:** General condition of animals at day 28.

Group	Eating	Drinking	Mental condition	Hair loss	Body weight
C	Normal	Normal	Normal	No	Normal
M	Normal	Normal	No obvious change	Heavy	Decreased
*M* + *Z*	Reduced	Reduced	Relatively poor	Alleviated Vs. M group	Decreased
*M* + *S*	Reduced	Reduced	Poor	Alleviated	Decreased
*M* + *Z* + *S*	Reduced	Reduced	Poor	Alleviated	Decreased

**Table 5:** The urine albumin/creatine ratio (mg/mg) in different groups ($\bar{x}\pm s$).

Time after adriamycin	Urine albumin/creatine (mg/mg)				
	C	M	*M* + *Z*		*M* + *Z* + *S*
Week 0	1.69 ± 0.75				
Week 1	1.63 ± 0.49	2.13 ± 0.25^★^	1.82 ± 0.25	1.69 ± 0.38^▲^	1.64 ± 0.23^▲^
Week 2	1.67 ± 0.40	2.19 ± 0.33^★^	2.02 ± 0.23	1.87 ± 0.12^▲^	1.82 ± 0.06^▲^
Week 3	1.63 ± 0.32	2.30 ± 0.28^★^	2.02 ± 0.09^▲^	1.97 ± 0.04^▲^	1.86 ± 0.21^#^
Week 4	1.64 ± 0.67	2.54 ± 0.39^★^	2.06 ± 0.21^▲^	2.04 ± 0.07^▲^	1.93 ± 0.09^#^

Control group (C), adriamycin group (M), adriamycin + Nephropathy Prescription I group (*M* + *Z*), adriamycin + prednisone acetate group (*M+S*), adriamycin + Nephropathy Prescription I + prednisone acetate group (*M* + *Z* + *S*). ^★^M vs. control group at the same time point (*P* < 0.05); ^▲^*M + S* vs. *M* group at the same time point (*P* < 0.05); ^#^*M* + *Z* + *S* vs. M group at the same time point (*P* < 0.01).

**Table 6:** Changes in the serum albumin content in different groups ($\bar{x}\pm s$).

Group	*N*	Albumin (g/L)
*C*	6	31.14 ± 3.66
*M*	6	25.38 ± 5.53^*∗*^
*M* + *Z*	6	25.32 ± 2.89
*M + S*	6	31.53 ± 4.12^&^
*M* + *Z* + *S*	6	32.68 ± 4.38^#^

Control group (C), adriamycin group (M), adriamycin + Nephropathy Prescription I group (*M* + *Z*), adriamycin + prednisone acetate group (*M+S*), adriamycin + Nephropathy Prescription I + prednisone acetate group (*M* + *Z* + *S*). ^*∗*^M vs. C group (*P* < 0.05); & *M + S* vs. *M* group (*P* < 0.05); ^#^*M* + *Z* + *S* vs. M group (*P* < 0.01).

**Table 7:** Changes in total cholesterol and triglyceride levels in different groups (mmol/L) ($\bar{x}\pm s$).

Group	N	TC (mmol/L)	TG (mmol/L)
*C*	6	2.84 ± 0.44	1.73 ± 0.25
*M*	6	6.42 ± 1.39^*∗*^	3.76 ± 0.94^*∗*^
*M* + *Z*	6	5.27 ± 1.03	2.72 ± 0.81^&^
*M* + *S*	6	3.23 ± 0.44^&^	2.34 ± 0.59^&^
*M* + *Z* + *S*	6	3.02 ± 0.37^&^	1.84 ± 0.68^#^

Control group (C), adriamycin group (M), adriamycin + Nephropathy Prescription I group (*M* + *Z*), adriamycin + prednisone acetate group (*M+S*), adriamycin + Nephropathy Prescription I + prednisone acetate group (*M* + *Z* + *S*). ^*∗*^M vs. C group (*P* < 0.05); ^&^*M* + *Z* or *M* + *S* vs. *M* group (*P* < 0.05); ^#^*M* + *Z* + *S* vs. M group (*P* < 0.01).

## Data Availability

The data used to support the article are included within the article.
