# Erratum to “Effect of Nephropathy Prescription I on the Expression of Angptl3 and Podocyte-Associated Protein in Mice with Adriamycin-Induced Nephropathy”

**DOI:** 10.1155/2023/9769430

**Published:** 2023-02-04

**Authors:** Feifei Zhang, Junchao Liu, Jian Yu, Wen Sun, Yonghong Wang, Teng Fan, Yanyan Sun, Xinghui Han

**Affiliations:** Children's Hospital of Fudan University, Department of Traditional Chinese Medicine, Shanghai 201102, China

In the article titled “Effect of Nephropathy Prescription I on the Expression of Angptl3 and Podocyte-Associated Protein in Mice with Adriamycin-Induced Nephropathy” [1], there was an error in Table 5. The corrected table is shown below and is listed as Table 5.

The error was introduced during the production process of the article, and Hindawi apologises for causing this error in the article.

## Figures and Tables

**Table 5:** The urine albumin/creatine ratio (mg/mg) in different groups (*x* ± *s*).

Time after adriamycin	Urine albumin/creatine (mg/mg)				
	*C*	*M*	*M* + *Z*	*M* + *S*	*M* + *Z* + S
Week 0			1.69 ± 0.75		
Week 1	1.63 ± 0.49	2.13 ± 0.25^★^	1.82 ± 0.25	1.69 ± 0.38^▲^	1.64 ± 0.23^▲^
Week 2	1.67 ± 0.40	2.19 ± 0.33^★^	2.02 ± 0.23	1.87 ± 0.12^▲^	1.82 ± 0.06^▲^
Week 3	1.63 ± 0.32	2.30 ± 0.28^★^	2.02 ± 0.09^▲^	1.97 ± 0.04^▲^	1.86 ± 0.21^#^
Week 4	1.64 ± 0.67	2.54 ± 0.39^★^	2.06 ± 0.21^▲^	2.04 ± 0.07^▲^	1.93 ± 0.09^#^

Control group (*C*), adriamycin group (*M*), adriamycin + nephropathy prescription I group (*M* + *Z*), adriamycin + prednisone acetate group (*M* + *S*), and adriamycin + nephropathy prescription I + prednisone acetate group (*M* + *Z* + S). ^★^*M* vs. control group at the same time point (*P* < 0.05), ^▲^*M* + *S* vs. *M* group at the same time point (*P* < 0.05), and ^#^*M* + *Z* + S vs. *M* group at the same time point (*P* < 0.01).
